# Subchondral Insufficiency Fracture of the Femoral Head Caused by Excessive Lateralization of the Acetabular Rim

**DOI:** 10.1155/2016/4371679

**Published:** 2016-05-11

**Authors:** Tetsuya Kimura, Tomohiro Goto, Daisuke Hamada, Takahiko Tsutsui, Keizo Wada, Shoji Fukuta, Akihiro Nagamachi, Koichi Sairyo

**Affiliations:** Department of Orthopedics, Institute of Biomedical Sciences, Tokushima University, 3-18-15 Kuramoto, Tokushima 770-8503, Japan

## Abstract

We present a case of a 53-year-old woman with subchondral insufficiency fracture (SIF) of the femoral head without history of severe osteoporosis or overexertion. Plain radiographs showed acetabular overcoverage with excessive lateralization of the acetabular rim. A diagnosis of SIF was made by typical MRI findings of SIF. The lesion occurred at the antipodes of the extended rim. Increased mechanical stress over the femoral head due to impingement against the excess bone was suspected as a cause of SIF. The distinct femoral head deformity is consistent with this hypothesis. This is the first report of SIF associated with acetabular overcoverage.

## 1. Introduction

Subchondral insufficiency fracture (SIF) of the femoral head is a rare injury with an uncertain etiology. It primarily occurs in elderly women with poor bone quality and occasionally in young active patients as a fatigue fracture [1–4]. SIF is typically seen in the anterosuperior portion of the femoral head corresponding to the weight bearing surface. Magnetic resonance imaging (MRI) is a useful tool for the definitive diagnosis of SIF, which is seen as a low intensity band surrounded by bone marrow edema on the T1-weighted image [1, 3]. Although it has been reported that acetabular dysplasia may contribute to the occurrence of SIF [5], there is no report of the cases with acetabular overcoverage associated with SIF. Here, we present a rare case of SIF possibly caused by mechanical overloading on the femoral head due to excessive lateralization of the acetabular rim, where the SIF lesion was posterolateral to the femoral head, facing the extended rim.

## 2. Case Report

**Figure 1:**
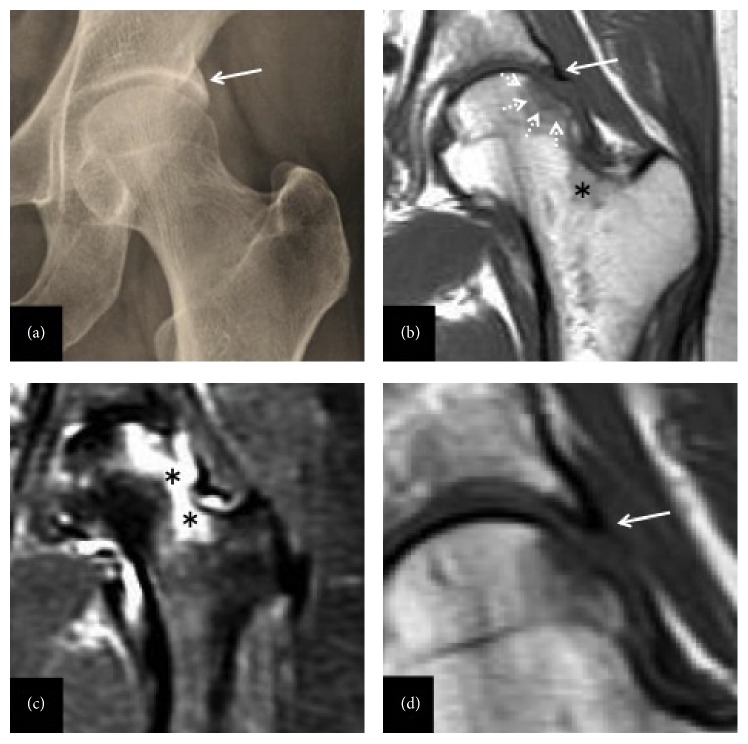
Initial radiograph and magnetic resonance imaging (MRI) of the left hip. (a) Anteroposterior view of the left hip shows acetabular overcoverage due to extended lateralization of the acetabular margin (white arrow). (b) T1-weighted image shows bone marrow edema with diffuse low intensity expanding from the lateral aspect of the femoral head to the intertrochanteric area. An irregular, serpentine, low intensity band (white dotted arrow) is present in the subchondral area. (c) Short-tau inversion recovery (STIR) image shows high signal intensity corresponding to the area of low intensity in (b). Black asterisks indicate the bone marrow edema of the femoral head and intertrochanteric area. (d) No apparent labral tear at the extended rim was observed but the labrum was relatively small and thin on the T1-weighted image (white arrow).

A healthy 53-year-old Japanese woman visited our hospital with acute onset of left hip pain. She was an elementary school teacher with no history of trauma, overexertion, corticosteroid intake, or alcohol abuse. She just had a physical education class as routine work a day before the onset of left hip pain. Physical examination revealed pain-induced limitation of the range of motion (ROM) of her left hip, with 90° flexion, 30° abduction, 20° adduction, 5° external rotation, and 15° internal rotation. The initial plain radiographs and computed tomography (CT) images of the left hip revealed a lateralized acetabular margin that extended laterally and distally from the posterolateral side of the acetabular rim (Figure 1(a)). A center-edge angle (CE angle) of 48° and an acetabular roof obliquity (ARO) of −9° indicated acetabular overcoverage. The patient had no other morphological abnormalities such as alpha angle (39.7°), acetabular version (29°), or antetorsion of the femoral neck (28°). Her posture was normal with pelvic tilt in both standing and supine positions of 10° forward to the anterior pelvic plane, using the bilateral anterior superior iliac spines and the top of the pubis as a reference plane. MRI revealed an irregular, serpentine, low intensity band surrounded by bone marrow edema at the subchondral area of the femoral head on T1-weighted images. The bone marrow edema was spreading from the lateral side of the femoral head to the trochanteric area (Figures 1(b) and 1(c)). No labral lesions were observed, although the labrum at the lateralized rim was noted to be small and thin (Figure 1(d)). The main lesion was observed at the antipodes of lateralized acetabular rim. The bone mineral density was relatively low (*T*-score: −1.3) but did not meet severe osteoporosis.

**Figure 2:**
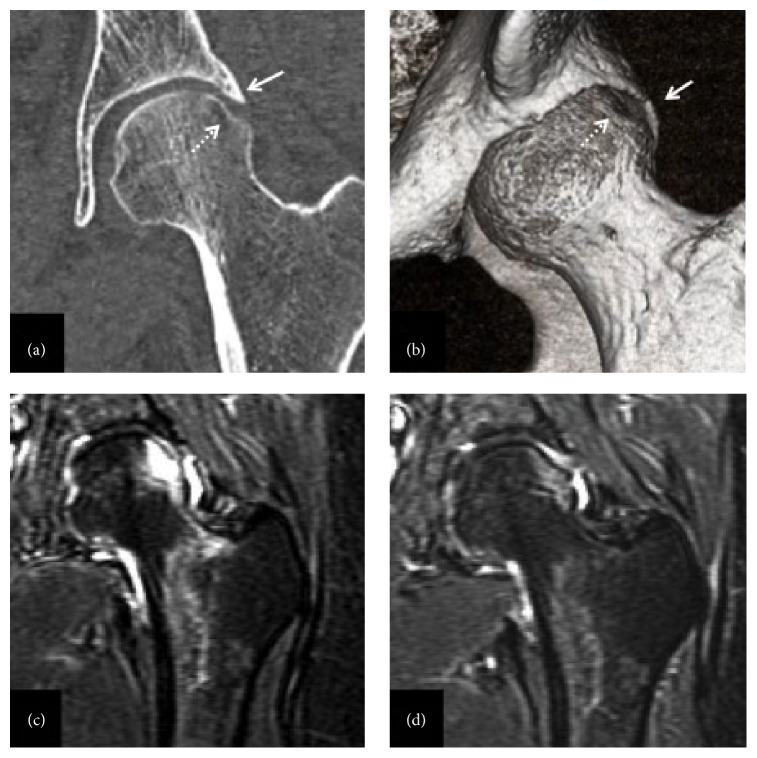
Partial collapse of the femoral head and serial MRI images. (a) Coronal computed tomography (CT) image after 4 weeks of therapy shows subchondral collapse (white dotted arrow) at the antipodes of the lateralized rim (white arrow). (b) A subchondral insufficiency fracture (SIF) is evident at the posterolateral aspect of the femoral head in the oblique three-dimensional CT view. STIR images show that the high intensity lesion (c) is smaller after 10 weeks of therapy and (d) has almost disappeared after 4 months of therapy.

**Figure 3:**
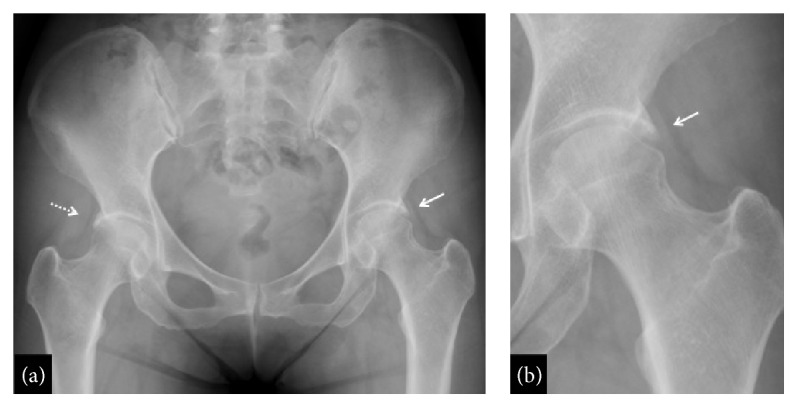
Plain radiographs at the last follow-up. (a) Anteroposterior X-ray obtained at the 2-year follow-up and (b) its enlarged image. Progression of the subchondral collapse was not observed but the acetabular overcoverage in both hips still remained (a dotted arrow indicates right and a white arrow indicates left side of the acetabular overcoverage).

SIF was diagnosed based on the typical MRI findings and she received conservative non-weight bearing treatment for 6 weeks using crutches. Subsequently, we allowed gradual partial weight bearing, one-third of her weight per week. Plain radiographs and CT images taken after 4 weeks of therapy showed partial collapse of the subchondral bone (Figures 2(a) and 2(b)). The collapse occurred as a surface imprint at the superolateral and relatively posterior aspects of the femoral head (Figure 2(b)). On MRI, the bone marrow edema was dramatically reduced and there was no advancement of the subchondral bone collapse (Figures 2(c) and 2(d)). Finally, the left hip pain disappeared entirely and she returned to work after 3 months of treatment. No recurrence of symptoms and no advancement of the subchondral bone collapse were observed at the 2-year follow-up (Figures 3(a) and 3(b)).

## 3. Discussion

SIF is a rare injury with an unknown etiology. By definition, SIF involves bone fragility secondary to osteoporosis or osteopenia leading to subchondral fractures in the femoral head with no evidence of osteonecrosis [2, 3, 6]. SIF has also been reported to occur as a fatigue fracture in young healthy patients [4]. In this paper, we present a case of SIF caused by acetabular overcoverage in a healthy (but slightly osteoporotic), middle-aged adult without evidence of overexertion.

Abnormalities are not generally evident on plain radiographs or CT in the early phase of SIF. Several months after onset, sclerotic changes due to fracture healing are sometimes observed in the subchondral area. MRI is the most useful modality for making a precise diagnosis. The characteristic MRI findings of SIF are diffuse bone marrow edema and a low intensity band on T1-weighted images. The shape of the low intensity band is typically discontinuous, irregular, serpentine, and parallel to the articular surface [1, 7]. This band lesion is the most distinctive finding for distinguishing SIF from other diseases. Differentiation of SIF from osteonecrosis of the femoral head (ONFH) or transient osteoporosis of the hip (TOH) is usually difficult and differentiating between these conditions is crucial because each condition has a different treatment and prognosis. ONFH is a progressive clinical condition with poor prognosis caused by ischemia of the femoral head, whereas TOH is characterized by temporal bone loss of the femoral head with unknown etiology and has a good prognosis with conservative treatment. Plain radiographs in both ONFH and TOH, especially in the early phase, usually show no apparent abnormalities so MRI is useful for making an early differential diagnosis. The common MRI finding in SIF, ONFH, and TOH is a bone edema pattern, which is defined as an area of low intensity on the T1-weighted image and high intensity on the T2-weighted and short-tau inversion recovery (STIR) images. In ONFH, a typical low intensity band on the T1-weighted image, which has a smooth, well delineated, and concave shape, is observed [7]. The features of this low intensity band are critical for differentiating between ONFH and SIF. On the other hand, in TOH, the highly suggestive MRI finding is a diffuse edema pattern without focal defects and subchondral changes, and these features resolve after treatment [8]. In the present case, MRI showed a low intensity band, an irregular, serpentine band surrounded by bone marrow edema at the subchondral area of the femoral head on the T1-weighted image, which was a typical feature of SIF. We could therefore exclude ONFH and TOH.

Although our patients showed typical characteristics of SIF on MRI, there was a clear difference from conventional SIF with respect to the location of the main lesion. In conventional cases of SIF, the main lesion tends to be observed at the anterosuperior portion of the femoral head [9]. In our case, they were observed in the posterolateral portion facing the excessively lateralized acetabular margin in the neutral hip position. MRI revealed that the lesions spread evenly from the lateral to posterolateral aspect of the femoral head. Given these findings, we hypothesize that increased mechanical stress over the femoral head due to impaction against the excessively lateralized rim caused SIF during weight bearing. The obvious femoral head deformity facing the extended rim is consistent with our hypothesis.

Recently, femoroacetabular impingement (FAI) has attracted attention as a cause of hip disorder. FAI involves abnormal abutment of the femur and acetabulum. It can be divided into two subtypes based on morphological abnormalities: cam and pincer. Asphericity of the femoral head causes cam impingement, whereas pincer impingement is a pathological condition caused by acetabular overcoverage [10]. Typical pincer impingement usually occurs during hip motion, especially in flexion with adduction and internal rotation or in extension with abduction and external rotation. The predominant injury pattern seen in pincer impingement involves the labrum at the anterior aspect of the joint and head-neck junction of the femur [11]. In our case, the bony protrusion of the acetabulum was seen from the lateral to posterior aspect, not at the anterior aspect, and the femoral lesion was seen at the posterolateral aspect of the femoral head, not at the femoral head-neck junction. Therefore, we suspect a pathology that was different from typical pincer impingement. Moreover, recent reports have shown atypical forms of hip impingement such as subspine impingement, ischiofemoral impingement, iliopsoas impingement, abnormal femoral antetorsion, and abnormal pelvic tilt [12, 13]. The first three types of atypical impingement (i.e., subspine impingement, ischiofemoral impingement, and iliopsoas impingement) have distinctive imaging findings, including bony protrusion of the anterior inferior iliac spine, a distinct pattern of diffuse edema of the quadratus femoris muscle, and an anterior labral tear adjacent to the iliopsoas tendon, respectively. In regard to the latter two types, our case had a normal range of femoral antetorsion and no significant change in pelvic tilt between supine and standing position; therefore, there is no evidence to associate the present case with atypical forms of hip impingement.

Although the inverted acetabular labrum was proposed to be the trigger of SIF [14], we could not detect an inverted labrum on MRI in our case. There was no apparent labral tear at the extended rim but the labrum was relatively small and thin (Figure 1(d)). We assume that such labral changes led to loss of the shock-absorbing function and increased mechanical stress over the femoral head during weight bearing. Although the present case does not provide sufficient evidence to support our hypothesis, a unique mechanism might have been involved in the pathology of SIF.

In the present case, early diagnosis of SIF was possible because MRI was performed at the initial consultation. Subchondral collapse was observed during treatment; however, we could prevent further progression of the collapse by an early and appropriate load relief on the femoral head. Although conservative therapy appeared to be successful in our case, the patient should be closely followed up to assess further pathological changes because the morphological abnormality such as acetabular overcoverage in both hips still remained (Figures 3(a) and 3(b)). We suggest the use of MRI for early diagnosis in patients with hip pain and ROM limitation even if they are middle-aged patients with no history of overexertion. Special attention should be given to patients who have acetabular overcoverage due to excessive lateralization of the acetabular rim, which may be a risk factor for SIF.
